# The Influence of Deleterious Mutations on Adaptation in Asexual Populations

**DOI:** 10.1371/journal.pone.0027757

**Published:** 2011-11-14

**Authors:** Xiaoqian Jiang, Zhao Xu, Jingjing Li, Youyi Shi, Wenwu Wu, Shiheng Tao

**Affiliations:** 1 Bioinformatics Center, College of Life Science, Northwest A&F University, Yangling, Shaanxi, China; 2 College of Science, Northwest A&F University, Yangling, Shaanxi, China; 3 School of Pharmacology, Shanghai Jiao Tong University, Shanghai, China; 4 State Key Laboratory of Crop Stress Biology for Arid Areas, Northwest A&F University, Yangling, Shaanxi, China; University of Utah, United States of America

## Abstract

We study the dynamics of adaptation in asexual populations that undergo both beneficial and deleterious mutations. In particular, how the deleterious mutations affect the fixation of beneficial mutations was investigated. Using extensive Monte Carlo simulations, we find that in the “strong-selection weak mutation (SSWM)” regime or in the “clonal interference (CI)” regime, deleterious mutations rarely influence the distribution of “selection coefficients of the fixed mutations (SCFM)”; while in the “multiple mutations” regime, the accumulation of deleterious mutations would lead to a decrease in fitness significantly. We conclude that the effects of deleterious mutations on adaptation depend largely on the supply of beneficial mutations. And interestingly, the lowest adaptation rate occurs for a moderate value of selection coefficient of deleterious mutations.

## Introduction

The appearance of beneficial mutations as well as their subsequent spread determines the adaptive process of a population. Generally speaking, a single beneficial mutation with small selection coefficient *s_b_*, has a fixation probability equal to approximately 2*s_b_*, where beneficial mutations are rare and get fixed independently [1]–[3]. However, a large supply of beneficial mutations does not result in a sequential fixation process in asexual populations [1], [4], [5]. There are two important processes affect the ability of asexual populations to accumulate beneficial mutations [6]. First, clonal interference (CI), the competition among two or more beneficial mutations from different lineage, leads to fixation of the best mutation and loss of the others [7]. Second, multiple mutations that are fixed simultaneously cause the lineage with a single mutation of large effect to be outcompeted by the lineage with several moderate effects mutations [8]. The CI theory has been demonstrated by both microbe experiments [9]–[12] and theoretical analyses [13]–[15]. But the experiment on asexual budding yeast that evolves in glucose-limited media supports the theoretical analysis of multiple mutations, i.e., the adaptation is dominated by the accumulation of multiple mutations with moderate beneficial effects [16].

Despite research efforts, the adaptation of asexual populations remains elusive. Almost all recent theoretical investigations focus on beneficial mutations, while the role of deleterious mutations in adaptation was neglected. Actually, deleterious mutations occur more frequently than beneficial ones in nature. If one beneficial mutation arises in a genetic background already carrying some deleterious mutations, their corresponding probability of fixation is reduced substantially [17]–[19]. Therefore, in a complete picture of adaptation, deleterious mutations should be taken into account. In the absence of Muller's ratchet, two possible scenarios are been considered to explain the influence of deleterious mutations on the population. Firstly, when the effects of beneficial mutations are smaller than the accumulated effects of deleterious ones, the latter are unlikely to spread, and adaptation is essentially constrained to beneficial mutations free of deleterious ones [19], [20]. Secondly, when the effects for beneficial mutations is larger than for deleterious ones, there is a chance for deleterious mutations to be fixed through hitchhiking with beneficial mutations, and meanwhile, the fixation chance for beneficial mutations increases accordingly [5], [21]–[24].

The above scenarios provide essential insights into the process of adaptation in asexuals, however, previous studies are limited in the cases that only one beneficial mutation get fixed in each fixation event. Once multiple beneficial mutations are allowed, how deleterious mutations put impact on the fixation process of beneficial mutations? Under what kind of conditions deleterious mutations would accumulate in adaptive process? To address these problems, we focus our attention on the distribution of selection coefficients of the fixed mutations (SCFM) and the number of mutations accumulated in a single fixation event. Moreover, we also estimate the adaptation rate in the long term evolution. Monte Carlo simulations, in combination with theoretical analyses, are conducted to explore adaptation process of asexual populations that subjects to both deleterious and beneficial mutations.

## Methods

### Model

Our simulation work is based on the Wright-Fisher model of asexually reproducing populations with fixed population size *N*. Each individual *i* in the population is initially assigned the identical fitness (*w_i,0_* = 1.0). The total mutation rate per genome per generation is *U* (*U* = *U_b_*+*U_d_*), where deleterious and beneficial mutation rate is *U_d_* and *U_b_*, respectively. We assume that the selection coefficients of both beneficial mutations (*s_b_*) and deleterious mutations (*s_d_*) are drawn from the following exponential distributions
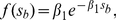
(1)

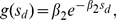
(2)And we use 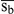 = 1/*β_1_* and 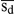 = 1/*β_2_* to separately represent the mean value of *s_b_* and *s_d_*. The exponential distribution of *s_b_* is supported by the extreme value theory [25]–[27]. Inasmuch as there is no generally accepted distribution of *s_d_*, we follow the study by Wilke (2004) to carry out our simulations with an exponential distribution truncated with the value of 1.0, which is necessary to avoid producing a negative fitness [20], [28]–[31].

We assume that all mutations act multiplicatively. A deleterious (or beneficial) mutation changes the relative fitness by a factor of 1-*s_d_* (or 1+*s_b_*) regardless of its genetic background. Hence, if the number of beneficial (or deleterious) mutations that an individual *i* carries is *m_b_* (or *m_d_*), we can calculate its fitness, *w_i_*, as follows:
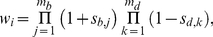
(3)where *s_b,j_* and *s_d,k_* are drawn from exponential distributions in Equation (1) and (2). A fixation event is defined as the first time when all individuals in population share a common ancestor. The selection coefficient of the common ancestor is *w_i_*-1, where *w_i_* is calculated by Equation (3). Here, we define *s_fix_* as the mean value of *w_i_*-1 in *M* repetitive simulations:
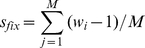
(4)


### Numerical simulations

In each generation, the number of new mutations occurring in an individual follows a Poisson distribution with mean *U*. Offspring are sampled with replacement according to a multinomial distribution, weighted by the fitness of their parent. Each individual *i* at generation *t+*1 is the offspring of an individual *j* at generation *t* with probability
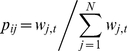
(5)During replication, the above mutation and selection steps are repeated until the occurrence of a single fixation event. We record the sum number of the fixed beneficial (or deleterious) mutations in 1,000 repetitive simulations, *n_b_* (or *n_d_*), and the mean selection coefficient of the common ancestor, *s_fix_*.

To estimate the average substitution rate of beneficial (or deleterious) mutations, E[*k_b_*] (or E[*k_d_*]), we run another group of simulations by 100 times, which sets the observation time up to 30,000 generations. We trace all the number of the accumulated beneficial and deleterious mutations in each simulation, and their corresponding fitness for obtaining an estimation for the change in log fitness over time (dlog*w*(*t*)/d*t*).

## Results

### The adaptive dynamics in a single fixation event

#### The distribution of SCFM

For any generated beneficial mutations with selection coefficient *s_b_*, the distribution of selection coefficients for the beneficial mutation in the presence of deleterious mutations that survive drift can be described as [11], [14]

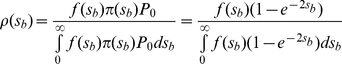
(6)where *π*(*s_b_*) is the probability of fixation of the beneficial mutation, and *P_0_* is the proportion of a population free of deleterious mutations. The denominator represents the average probability of surviving drift across the distribution of beneficial selection coefficient [14]. We use the expression 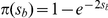, which remains valid for large *s_b_* [32]. If *Ns_d_*≫1, the frequency of deleterious mutations in a finite population follows a Poisson distribution with mean value *U_d_/s_d_*. The fraction of free of deleterious mutations is very close to that expected for an infinite population, 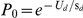 [33].

In the simple SSWM regime where beneficial mutations are rare and get fixed in succession, the distribution of SCFM, 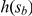, is expected to be equal to 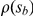. If *s_b_* is small, 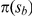 is roughly equal to 2*s_b_*. In such case, 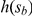 follows a Gamma distribution with shape parameter 2. Once clonal interference occurs, the fixation probability of a beneficial mutation is reduced by a factor of 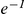 with
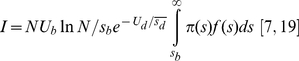
(7)and the distribution of SCFM becomes
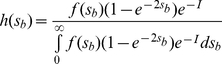
(8)As a common factor, 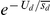 in the numerator and denominator in Equation (8) can be removed, which offsets the influence of deleterious mutations on 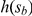. This indicates that deleterious mutations would not change the distribution of SCFM given one-by-one fixation of beneficial mutations in both the SSWM and CI regimes.

With the increasing supply of beneficial mutations, the adaptation depends on the interaction between both clonal interference and multiple mutations. And when both beneficial and deleterious mutations have a broad range of selection coefficient, it is difficult to obtain a precise prediction of 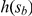. Here, we only display our simulation results in this complex regime.

**Figure 1:**
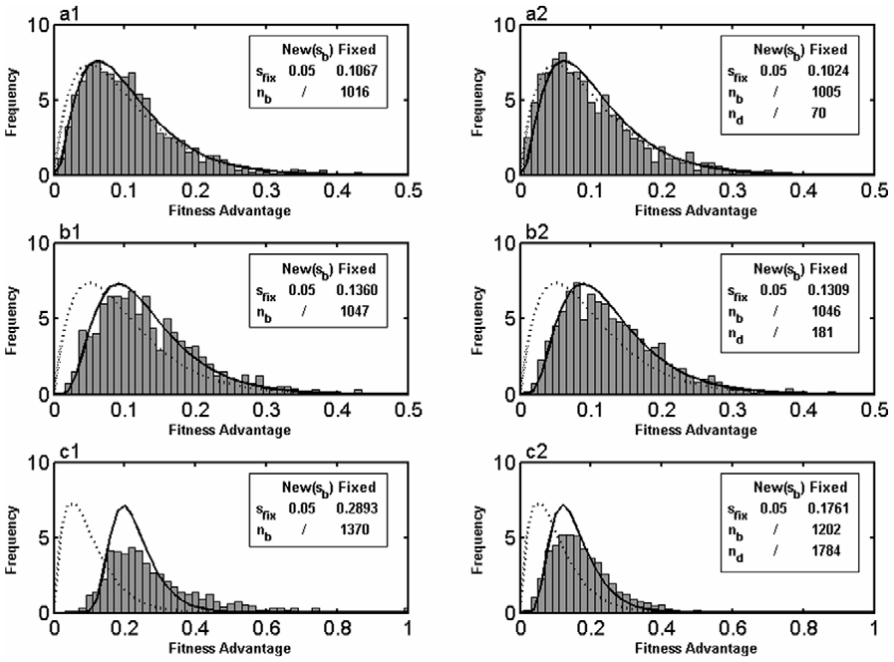
Examples of distribution of selection coefficients of fixed mutations. The distribution (*h*(*s*)) from our simulations (histogram) is compared with that deduced from SSWM analysis (dashed curve) and CI analysis (solid curve). In all simulations, *N* = 10^4^, *β_1_* = 20, *β_2_* = 10, *p_b_* = 0.001 (if deleterious mutations are included). a1, *U_b_* = 2.0×10^−6^, *U_d_* = 0. a2, *U_b_* = 2.0×10^−6^, *U_d_* = 2.0×10^−3^. b1, *U_b_* = 1.0×10^−5^, *U_d_* = 0. b2, *U_b_* = 1.0×10^−5^, *U_d_* = 1.0×10^−2^. c1, *U_b_* = 2.0×10^−4^, *U_d_* = 0. c2, *U_b_* = 2.0×10^−4^, *U_d_* = 2.0×10^−1^.

In Figure 1, we show several examples of the distribution of SCFM, 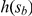, as compared to the SSWM prediction by Equation (5) and the CI prediction by Equation (7), respectively. In the case of a low *U_b_*, where neither clonal interference nor multiple mutations occurs, the SSWM analysis could give an accurate description of 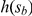 (Figure 1.a1). While with a moderate value of *U_b_*, clonal interference works and only the fittest mutation could be fixed in the population (Figure 1.b1). In both the SSWM and CI regimes, deleterious mutations hardly influence the distribution of SCFM, which is well consistent with the CI prediction (Figure 1.a2 and b2). Although a few deleterious mutations get fixation due to the potential distribution of *s_d_*, their effects on the distribution of SCFM can be safely ignored.

With a high input of beneficial mutations, it becomes more likely for multiple mutations to arise from the same background. We have observed that the effects of multiple mutations on the statistics of substitution events is important, causing 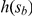 to deviate from the CI prediction apparently (Figure 1.c1). In such case, many deleterious mutations are fixed by linkage with beneficial mutations and *s_fix_* declines substantially (Figure 1.c2). In Table S1, all the fitness effects of each fixed mutation on the simulation results are presented (corresponding to Figure 1. c2). We can see that many fixed beneficial mutations occur in individuals with a few small effects deleterious mutations (the relative fitness effects of deleterious mutations are usually larger than 0.99). Although the total number of fixed beneficial mutations decreases, the fixation of beneficial mutations with large effects dominates over the accumulation of deleterious mutations by Muller's ratchet, resulting in an always positive fitness of the population.

#### Accumulated mutations in a single fixation event

**Figure 2:**
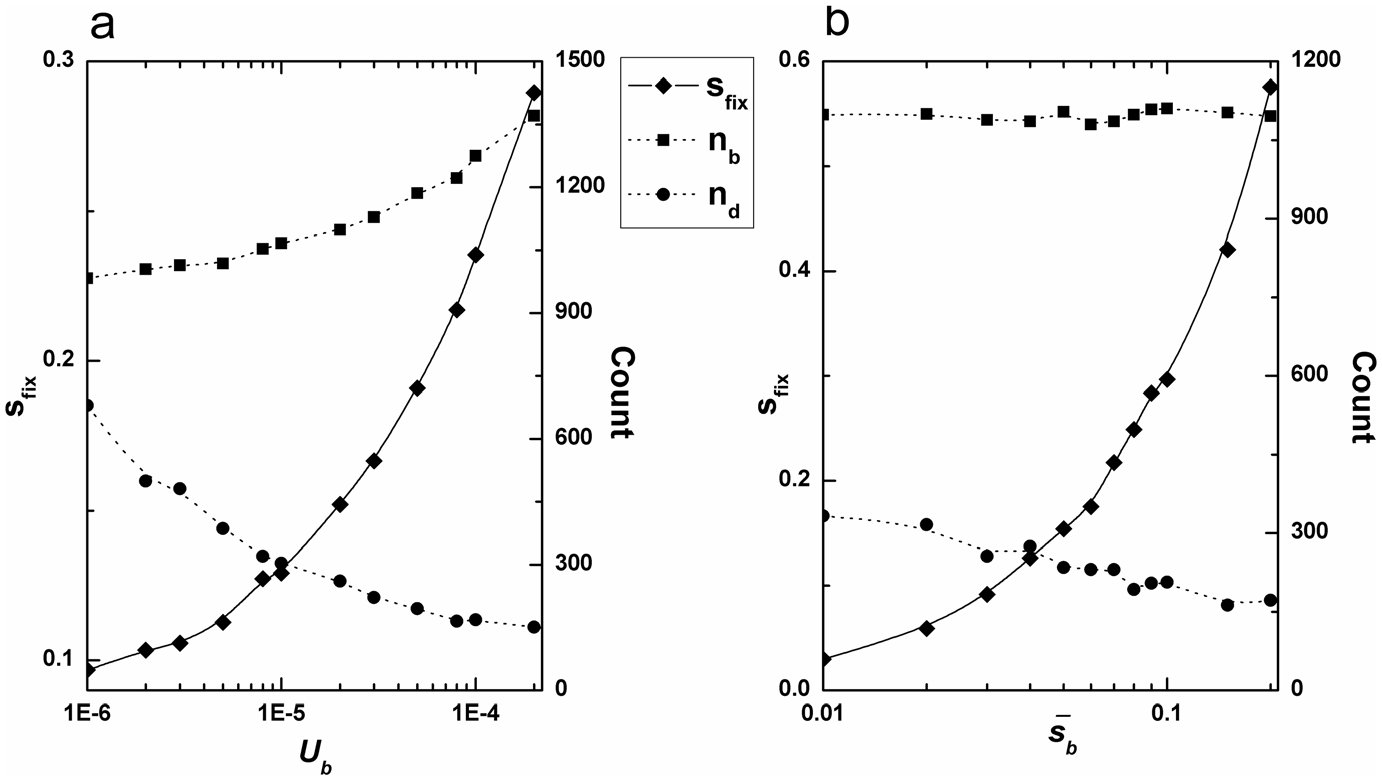
Changes in dynamics versus beneficial mutations. a, *s_fix_*, *n_b_*, and *n_d_* versus *U_b_* for *N* = 10^4^, *β_1_* = 20, *U_d_* = 1.0×10^−2^, *β_2_* = 20. (b), *s_fix_*, *n_b_*, and *n_d_* versus 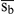 for *N* = 10^4^, *U_b_* = 2.0×10^−5^, *U_d_* = 1.0×10^−2^, *β_2_* = 20.

In Figure 2, we have plotted the adaptive dynamics in the first fixation event as a function of beneficial mutations, *U_b_* and 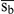, respectively. In these simulations, an increase in either *U_b_* or 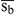 could results in a decrease in *n_d_* when the rate and mutational effects of deleterious mutations are constant. With the increase in the supply of beneficial mutations, both CI and multiple mutations could take place, making the necessary generations for a single fixation event shorter (data not shown). This reduces the fixation chance of deleterious mutations and thereby causes a decrease in *n_d_*.

**Figure 3:**
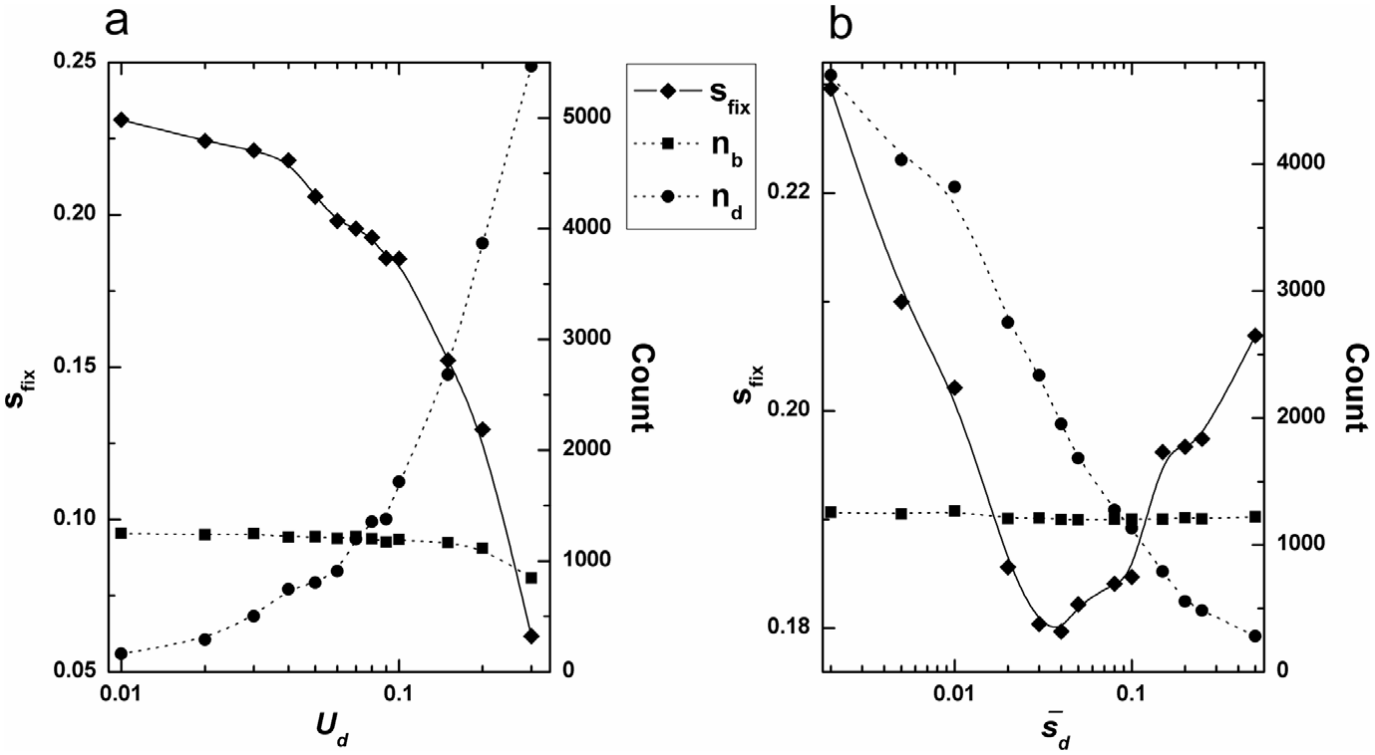
Changes in dynamics versus deleterious mutations. a, *s_fix_*, *n_b_*, and *n_d_* versus *U_d_* for *N* = 10^4^, *U_b_* = 1.0×10^−4^, *β_1_* = 20, *β_2_* = 20. b, *s_fix_*, *n_b_*, and *n_d_* versus 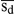 for *N* = 10^4^, *U_b_* = 1.0×10^−4^, *β_2_* = 20, *U_d_* = 0.1.

In Figure 3, we show the simulation results for *s_fix_*, *n_b_* and *n_d_* as a function of deleterious mutations, *U_d_* and 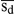, respectively for constant *U_b_* and 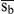. An increasing supply of deleterious mutations leads to no apparent change in *n_b_*. However, for very high values of *U_d_* (≈0.2), the fixation probability of beneficial mutations decreases drastically. At this point, a substantial fraction of fixation events contain net negative effects mutations, implying the operation of Muller's ratchet. We have observed a visibly different trend for *s_fix_* by changing 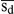, which reaches rock-bottom and rises up again with the increase in 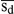. Note that the bottom point roughly corresponds to the situation where 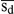 is roughly equal to 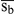 (≈0.05). If most beneficial mutations share the similar absolute fitness effects to deleterious mutations, the effects of deleterious ones that counteract beneficial ones reaches the maximum, which makes the results intuitively. As *s_d_* decreases, deleterious mutations are more likely to get fixation by linkage with beneficial mutations, but their effect in reducing the advantage of the beneficial -mutations is less. Therefore, we expected that, for some moderate values of 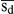, the maximum “dragged” effect of deleterious mutations will emerge.

### The rate of adaptation

In the presence of deleterious mutations, if beneficial mutations have independent fates, the substitution rate of beneficial mutations, *K_b_*, is defined as
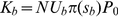
(9)Where 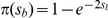 and 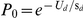 [19]. When clonal interference takes place, the probability of fixation is reduced by a factor of 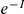, where *I* is determined by Equation (7). The CI theory assumes that only one beneficial mutation is fixed in each fixation event. Then, the expected average substitution rate of beneficial mutations becomes [7], [19], [20]

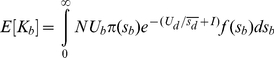
(10)And the mean selection coefficient of fixed mutations is
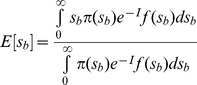
(11)where 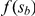 comes from Equation (1). Then the change in log fitness is predicted to be
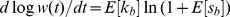
(12)


**Figure 4:**
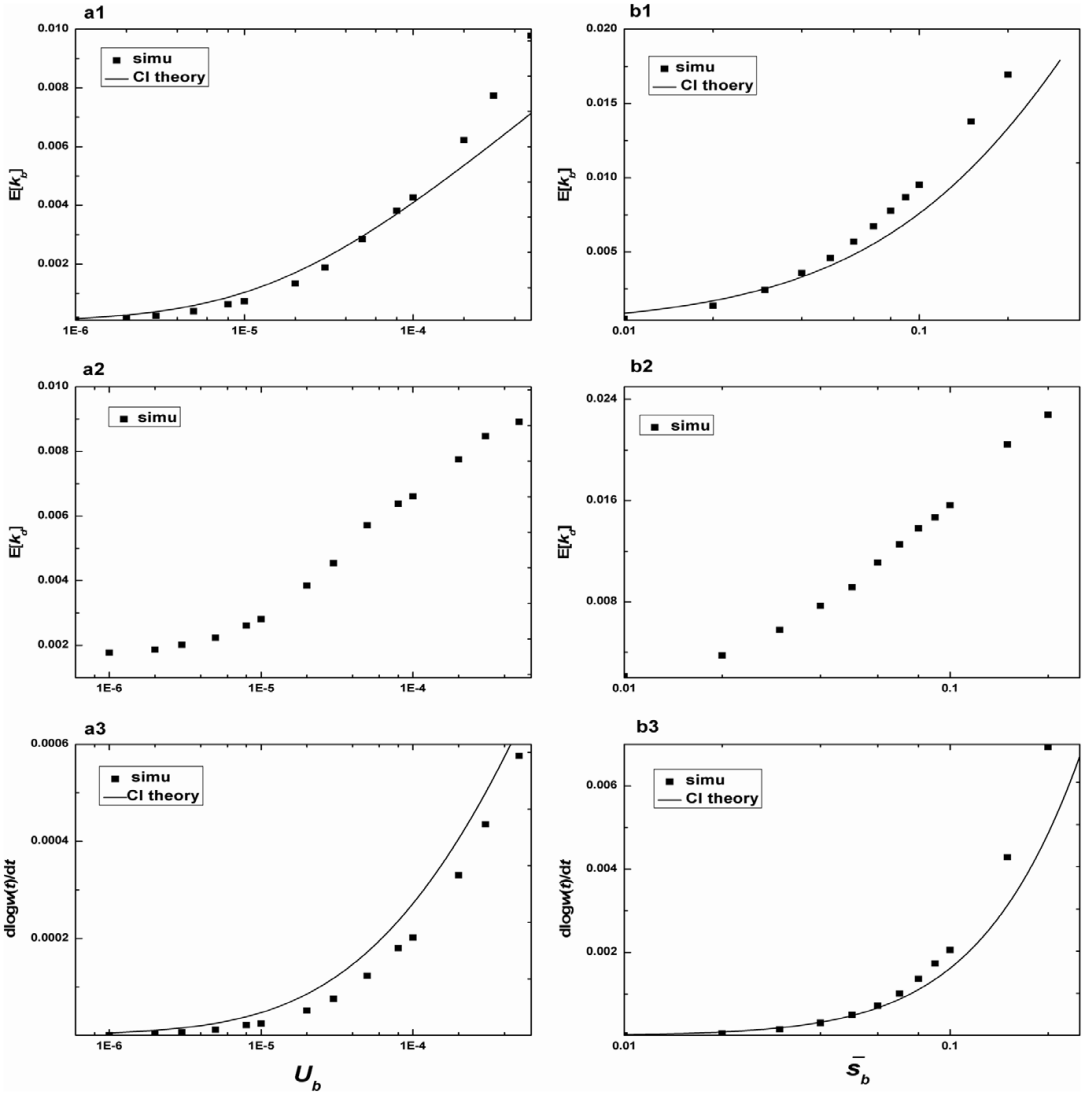
The substitution rate versus beneficial mutations. a, E[*k_b_*], E[*k_d_*], dlog*w*(*t*)/*dt* versus *U_b_* for *N* = 10^4^, *β_1_* = 50, *U_d_* = 1.0×10^−1^, *β_2_* = 10. b, E[*k_b_*], E[*k_d_*], dlog*w*(*t*)/*dt* versus
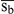
for *N* = 10^4^, *U_b_* = 2.0×10^−5^, *U_d_* = 1.0×10^−1^, *β_2_* = 10. Solid lines are theoretical predictions from Equation (9) (E[*k_b_*]) and Equation (11) (dlog*w*(*t*)/*dt*), and points are simulation results.

**Figure 5:**
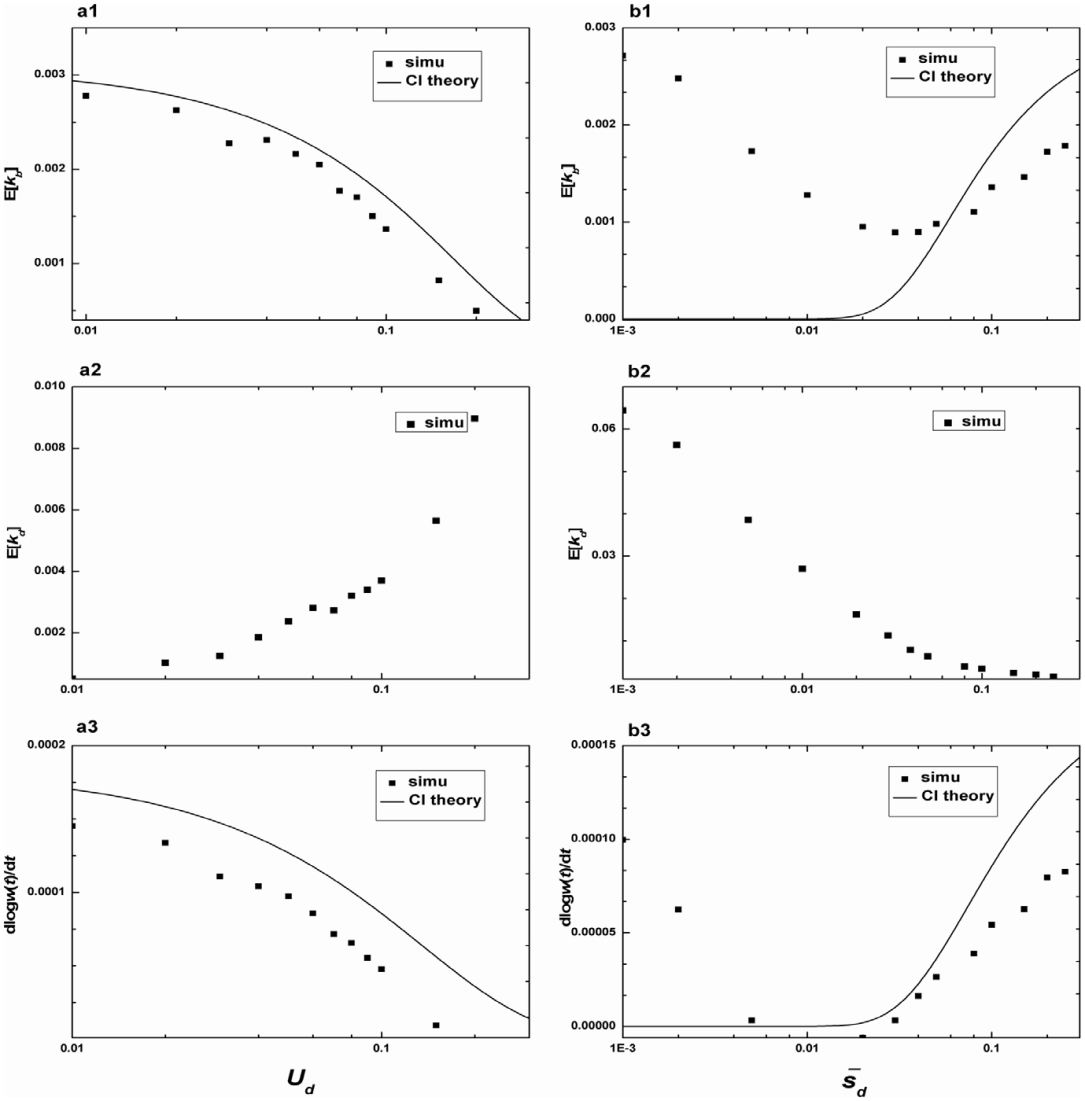
The substitution rate versus deleterious mutations. a, E[*k_b_*], E[*k_d_*], dlog*w*(*t*)/*dt* versus *U_d_* for *N* = 10^4^, *U_b_* = 2.0×10^−5^, *β_1_* = 50, *β_2_* = 10. b, E[*k_b_*], E[*k_d_*], dlog*w*(*t*)/*dt* versus 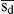 for *N* = 10^4^, *U_b_* = 2.0×10^−5^, *β_1_* = 50, *U_d_* = 1.0×10^−1^. Solid lines are theoretical predictions from Equation (9) (E[*k_b_*]) and Equation (11) (dlog*w*(*t*)/*dt*), and points are simulation results.

Figs. 4–5 compare our simulation results with the CI predictions. When examining the influence of one parameter on adaptation rate, we hold other parameters constant. In Figure 4, we show the average substitution rate (E[*k_b_*], E[*k_d_*], and dlog*w*(*t*)/*dt*) as a function of the input of beneficial mutations (*U_b_*, 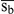). We have observed that with an increase in *U_b_*, the accumulation of both multiple beneficial mutations and slightly deleterious mutations that hitchhike with beneficial ones makes the CI theory to underestimate E[*k_b_*]. As shown above, the CI theory assumes that only those beneficial mutations free from deleterious mutations background could get fixed. However, for high value of *U_d_*, deleterious mutations occur so frequently that beneficial mutations occurring from deleterious background also get fixation. Note that the CI prediction underestimates both E[*k_b_*] and dlog*w*(*t*)/*dt* for large 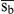. This phenomenon is mainly caused by the accumulation of the multiple beneficial mutations rather than by the fixation of deleterious mutations, because the fixation of large effects beneficial mutations is rarely influenced by slightly deleterious mutations. And the *U_b_* used here is large enough to cause the fixation of multiple beneficial mutations. By contrast, if *U_b_* is small and no multiple mutations occur, the CI analysis overestimates both E[*k_b_*] and dlog*w*(*t*)/*dt* due to the occurrence of slightly deleterious mutations (see Figure S1).

In Figure 5, two trends are worthy of comments. First, the CI theory always overestimates both E[*k_b_*] and dlog*w*(*t*)/*dt* as *U_d_* increases. Although multiple beneficial mutations occur frequently for *U_b_* = 2.0×10^−5^, their additive effects (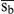 = 0.02) could not compensate for the “dragged” effect caused by deleterious mutations. However, we expect that if 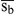 increases, the accumulation of multiple beneficial mutations with larger effects will make CI theory underestimate the adaptation rate (see Figure S2). Second, for some intermediate values of 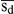, the “dragged” effect by deleterious mutations could achieve its maximum value, resulting in the lowest values of both E[*k_b_*] and dlog*w*(*t*)/*dt*. Note that, the population accumulates the net negative mean fitness (dlog*w*(*t*)/*dt*≈−3.23×10^−6^) given 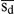 = 0.02, indicating that Muller's ratchet is the driving force in evolution.

## Discussion

We have presented a detailed study of the adaptive process in asexual populations by using extensive Monte Carlo simulation, where the population is subject to both beneficial and deleterious mutations. Taking account of mutational effects that vary across different loci in genome, the model has assumed that the selection coefficients (*s_b_* and *s_d_*) follow continuous exponential distributions [24], [34]–[36]. For instance, experiment in bacteria shows that adaptation is driven by high beneficial mutation rate (*U_b_*≈10^−5^) and small effects (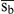
≈0.01) [12]. And the average beneficial effects in evolving *Pseudomonas fluorescens* population is in a very broad range from 0.023 to 0.089 [38]. The direct estimate for deleterious mutations in *vesicular stomatitis virus* shows 0.19 reduction of average fitness (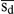
≈0.19) [36].

We have shown the effects of deleterious mutations on the distribution of SCFM (Figure 1). As demonstrated in previous studies, a continuous supply of deleterious mutations affects the fate of beneficial mutations in a subtle way [18], [21]–[23], [37]. According to deleterious mutation rate and their fitness effects, there are two different cases. First, if *U_d_/s_d_*<1, the subpopulation without deleterious mutations is larger than that with one deleterious mutation. It is easy to see that most of the fixed beneficial mutations arise from the background without deleterious mutations (Figure 1.a2 and b2). Second, if *U_d_/s_d_*>1, the situation becomes complicated and whether the mean fitness of the population will increase depends on the input of beneficial mutations. With rare beneficial mutations, Muller's ratchet will dominate and individuals initially with a net negative fitness also get fixed in the population. In this case, deleterious mutations will inevitably be accumulated, reducing mean fitness of the population evidently. By contrast, with high input of beneficial mutations, deleterious mutations can be fixed only by hitchhiking with beneficial mutations. It is likely that multiple beneficial mutations arise in such situation, making the fixed selection coefficients to be overestimated as compared to the prediction of CI theory. And beneficial mutations occurring in background with a few deleterious mutations might have a higher fitness than those in the background without deleterious mutations. In this case, fixation of deleterious mutation by hitchhiking with beneficial ones can frequently happen, which changes the statistics of the fixation and adaptive process (Figure 1.c2). Even though the fixation of beneficial mutations dominates over the action of Muller's ratchet, the fixation of large number of slightly-deleterious mutations reduces the fixed fitness largely.

Our results also illustrate that there exists the minimum mean fitness of the population as *s_d_* changes (for the special case of *s_d_*≈0.02 in Figure 5. b3). For some intermediate value of *s_d_*, the “dragged” effects caused by deleterious mutations put a significant impact on adaptive process. The reason is that deleterious mutations of large effects could be eliminated quickly by selection, and the accumulation of deleterious mutations with “nearly neutral” effects also contributes little to the net advantage fitness of the population. Thus in populations that deleterious mutations have some moderate effects, Muller's ratchet might dominate over the fixation of beneficial mutations, leading to the degeneration of the population.

Only when the selection on deleterious mutations is weaker than that on beneficial mutations, deleterious mutations are likely to have a chance to contribute to adaptation [24]. Hence, the fixed selection coefficients should be treated with caution because they might be composed of multiple beneficial and deleterious mutations rather than a single beneficial one. Although those strongly favored mutations increase the net fitness of the population largely in a narrow sense, they may cause an irreversible loss of gene functions due to the linkage of a large number of weakly deleterious mutations. This process may result in a long-term negative effects that offset the new beneficial mutations on the population [5], [39], [40]. A possible example relevant to this explanation is the degeneration of non-recombining Y chromosomes [41], [42].

Our studies have illustrated how the interplay between beneficial and deleterious mutations puts impact on the adaptive dynamics. Although we see that deleterious mutations reduce the population adaptation rate evidently, whether they could contribute to adaptation depends largely on the supply of beneficial mutations and the “dragged” effect is the largest when deleterious mutations have some moderate effects.

## Supporting Information

Figure S1 **The substitution rate (E[** ***k_b_*** **], E[** ***k_d_*** **], dlog** ***w*** **(** ***t*** **)/** ***dt*** **) versus** ***s_b_*** **for** ***N*** **= 10^4^,** ***U_b_*** **= 1.0×10^−5^,** ***U_d_*** **= 1.0×10^−1^,** ***β_2_*** **= 10.** Solid lines are theoretical predictions from Equation (9) (E[*k_b_*]) and Equation (11) (dlog*w*(*t*)/*dt*), and points are simulation results.
(TIF)

Figure S2 **The substitution rate (E[** ***k_b_*** **], E[** ***k_d_*** **], dlog** ***w*** **(** ***t*** **)/** ***dt*** **) versus** ***U_d_*** **for** ***N*** **= 10^4^,** ***U_b_*** **= 2.0×10^−5^,** ***β_1_*** **= 5,** ***β_2_*** **= 10.** Solid lines are theoretical predictions from Equation (9) (E[*k_b_*]) and Equation (11) (dlog*w*(*t*)/*dt*), and points are simulation results.
(TIF)

Table S1 **The fitness effects of each mutation fixed in 1,000 simulation results in** **Figure 1.c2** **.** The parameters used here: *N* = 10^4^, *β_1_* = 20, *β_2_* = 10, *U_b_* = 2.0×10^−4^, *U_d_* = 2.0×10^−1^. The fitness effect *w* is equal to 1+*s_b_* (or 1−*s_d_*). Therefore, *w*<1 stands for a deleterious mutations while *w*>1 stands for a beneficial mutation. The total fixed beneficial/deleterious mutation number is 1202/1784 and the mean fitness is 1.1761.
(XLS)
