# Supplementary figures and images for: The Influence of Deleterious Mutations on Adaptation in Asexual Populations
Source: PLoS One. 2011 Nov 14;6(11):e27757. doi: 10.1371/journal.pone.0027757 (PMC3215719; doi:10.1371/journal.pone.0027757)

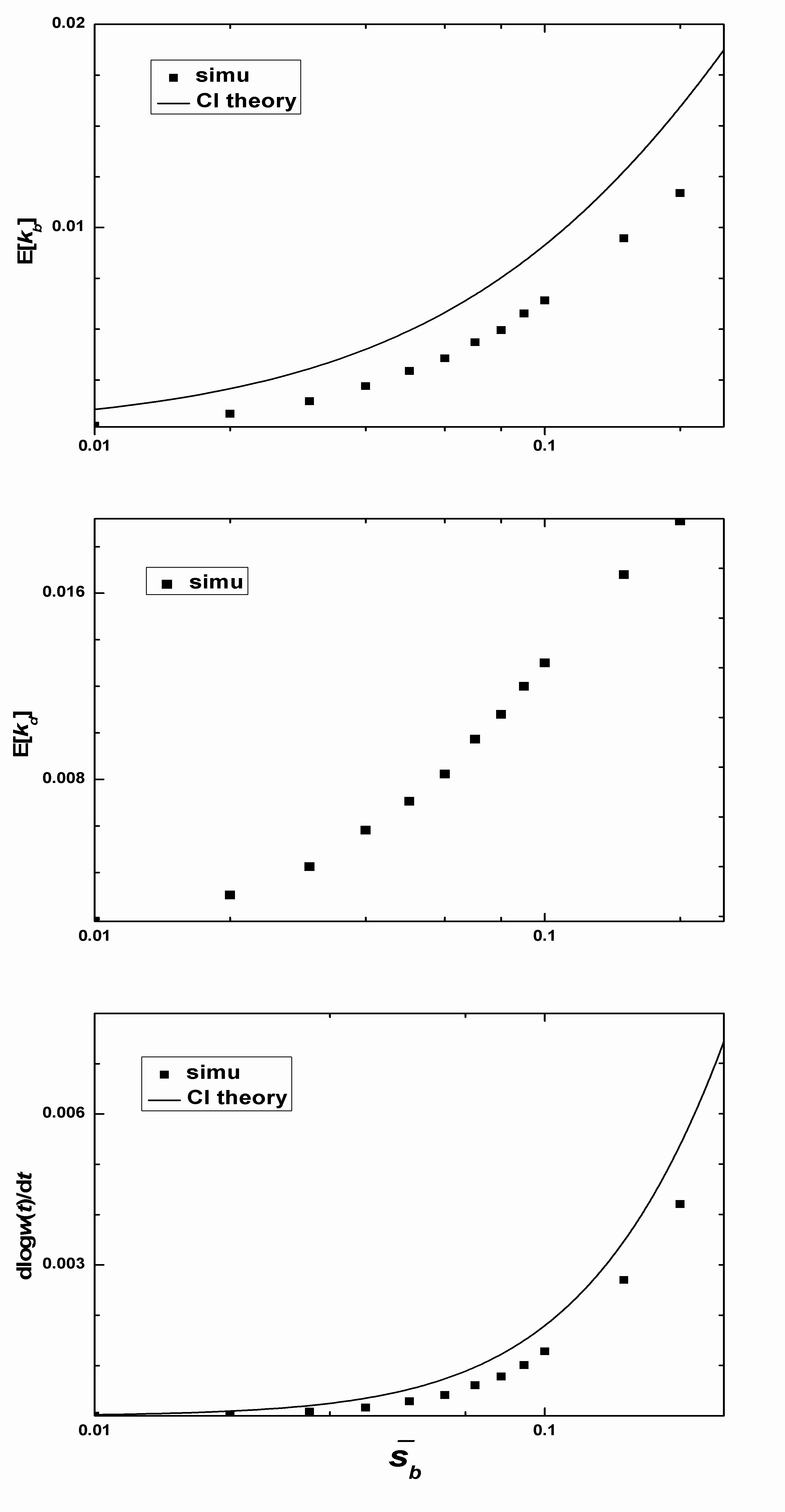

Supplement: Figure S1 — The substitution rate (E[ kb ], E[ kd ], dlog w ( t )/ dt ) versus sb for N = 104, Ub = 1.0×10^−5^, Ud = 1.0×10^−1^, β2 = 10. Solid lines are theoretical predictions from Equation (9) (E[kb]) and Equation (11) (dlogw(t)/dt), and points are simulation results. (TIF) [file pone.0027757.s001.tif]

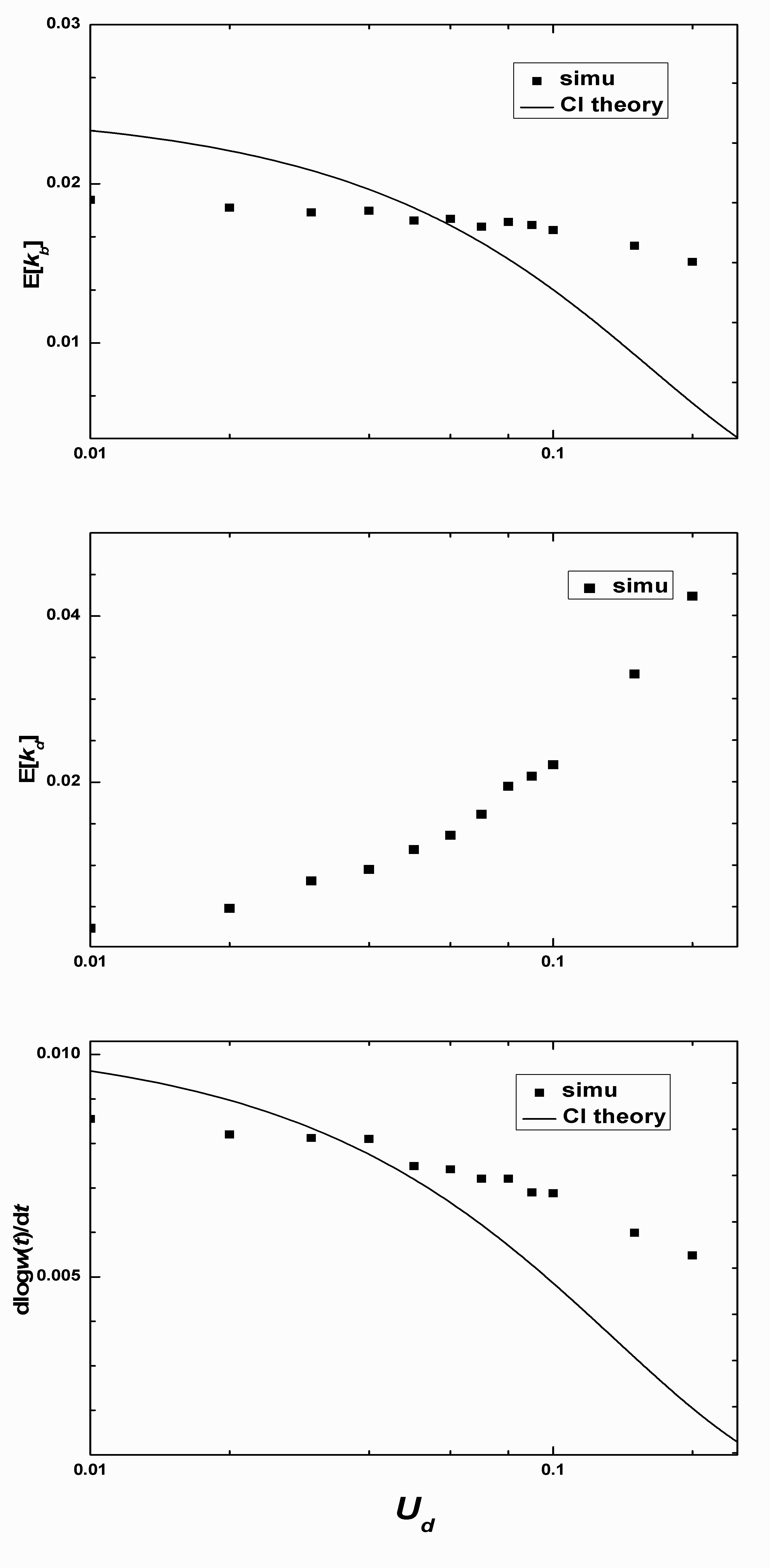

Supplement: Figure S2 — The substitution rate (E[ kb ], E[ kd ], dlog w ( t )/ dt ) versus Ud for N = 104, Ub = 2.0×10^−5^, β1 = 5, β2 = 10. Solid lines are theoretical predictions from Equation (9) (E[kb]) and Equation (11) (dlogw(t)/dt), and points are simulation results. (TIF) [file pone.0027757.s002.tif]
